# Comparison of invasive and non-invasive gradients before and after TAVI and their implications on clinical outcomes

**DOI:** 10.1007/s12928-024-01074-6

**Published:** 2024-12-19

**Authors:** Anna Pfenniger, Thorald Stolte, Jakob Johannes Reichl, Gregor Leibundgut, Max Wagener, Christoph Kaiser, Jasper Boeddinghaus, Felix Mahfoud, Thomas Nestelberger

**Affiliations:** 1 Department of Cardiology and Cardiovascular Research Institute Basel (CRIB), University Hospital Basel, University of Basel, Petersgraben 4, 4031 Basel, Switzerland; 2 Department of Health Sciences and Technology, Swiss Federal Institute of Technology, Zurich, Switzerland

**Keywords:** Transcatheter aortic valve implantation, Aortic stenosis, Transthoracic echocardiography, Transvalvular pressure gradient, Structural valve deterioration

## Abstract

Transcatheter aortic valve implantation (TAVI) is recommended for treatment of high-risk aortic stenosis patients. While measuring mean transaortic valve gradient (MG) is crucial in evaluating procedural success, echocardiographic measurements often overestimate direct invasive measurements. This study aimed to examine the discordance between echocardiographic and invasive MGs in TAVI patients and assess their prognostic value on long-term outcomes. This prospective registry included consecutive TAVI patients at a tertiary university hospital. Transthoracic or transoesophageal echocardiography was performed pre-TAVI, at discharge, 1 month, 1, and 5 years with invasive MG measurements obtained peri-procedurally. The primary endpoints were 5-year all-cause mortality and major adverse cardiac events. Among 1353 patients from 2011 to 2023, non-invasive MGs exceeded invasive MGs pre- and post-implantation (43 [36, 52] mmHg vs. 40 [30, 50] mmHg, *p* < 0.001; 9 [6, 12] mmHg vs. 4 [2, 7] mmHg, *p* < 0.001) Pre-procedural MGs correlated better than post-procedural MGs (*r* = 0.70, *p* < 0.001 vs. *r* = 0.23, *p* < 0.001), particularly in self-expandable valves and smaller sinus of Valsalva diameter (SOVd) (*r* = 0.33, *p* < 0.001; *r* = 0.46, *p* < 0.001 vs *R* = 0.06, *p* = 0.701). Non-invasive MG remained stable from discharge (9 [6, 12] mmHg) to 5 years (9 [7, 12] mmHg). While structural valve deterioration and patient-prosthesis mismatch showed no impact, extreme invasive MGs (< 3 or > 6 mmHg) predicted worse outcomes (mortality hazard: 1.25 [1.06, 1.88] and 0.85 [0.8, 0.95], respectively), unlike non-invasive measurements. In conclusion, invasive as compared with non-invasive MGs correlated better before than after valve implantation, whereas invasive MGs were always lower than non-invasive MGs. Lower invasive MGs after TAVI appeared to be associated with favourable long-term outcomes.

**Supplementary Information:**

The online version contains supplementary material available at 10.1007/s12928-024-01074-6.

## Introduction

Transcatheter aortic valve implantation (TAVI) is recommended for the treatment of severe aortic stenosis (AS) in symptomatic patients older than 75 years and for patients with high or prohibitive surgical risk [1, 2]. The transvalvular pressure gradient is a standard measure to assess stenosis severity and can be derived non-invasively from transaortic velocities using the simplified Bernoulli equation. Additionally, invasive measurements, such as peak to peak gradient (PG) and mean gradient (MG), can be obtained by introducing catheters into the left ventricle and the ascending aorta [3]. Both invasive and non-invasive MGs measured before TAVI demonstrate strong correlation and agreement, demonstrating their reliability in the assessment of native AS [4].

The success of TAVI, however, is mainly determined by post-procedural echocardiographic assessments, which evaluate the accurate position and function of the prosthetic valve including PG and MG as well as the extent of paravalvular leak. To standardize important clinical outcomes associated with bioprosthetic function, device success, and to facilitate comparisons among different clinical studies, the Valve Academic Research Consortium (VARC) consensus reports have been published [5]. The echocardiographic thresholds proposed by VARC-3 for assessing prosthetic valve performance after TAVI have been widely adopted [6–10]. Several studies have highlighted an overestimation of transvalvular MG by echocardiography compared to direct invasive measurements, particularly in the post-procedural setting. However, these studies had small sample sizes and lacked an assessment of long-term outcome [6, 7].

This study aimed to examine the discordance between echocardiographically derived and invasively obtained measurements of mean transaortic valve gradients in patients undergoing TAVI and their impact on long-term outcomes in an all-comer population at a tertiary hospital over the last 11 years.

## Methods

### Study design and patient cohort

Since September 2011, patients undergoing TAVI at the University Hospital Basel (USB), Switzerland have been included in the SwissTAVI registry (NCT01368250), a prospective national database. The study followed the principles of the Declaration of Helsinki and good clinical practice. The local ethics committee and all institutional review boards approved the registry. Data monitoring and endpoint adjudication was standardized by the VARC [10]. All patients enrolled provided written informed consent for study participation and prospective follow-up assessment. Prior results have been published based on the registry [11–14].

### Clinical data and follow-up

Baseline characteristics, peri-procedural, interventional, and follow-up information were collected using a standardized case report form and recorded in a web-based database. Clinical events occurring during the hospital stay or during follow-up were systematically collected and adjudicated by a dedicated clinical event committee following review of the original source documents. The responsibility for data monitoring, verification of completeness, and plausibility lies with the Clinical Trial Unit at the University of Bern.

### Echocardiography

All patients received a transthoracic (TTE) and/or transoesophageal (TEE) echocardiography by an experienced cardiologist or sonographer at baseline, post-procedural, and during follow-up using a commercially available ultrasound machine according to current guidelines [1, 2]. Left ventricular outflow tract (LVOT) diameter (LVOTD) was assessed prior to TAVI in a parasternal long-axis zoom view. PG and MG were assessed by placing the pulsed wave Doppler signal directly above the aortic valve leaflets or just above the prosthesis in subcostal views or in a parasternal right view, as close as possible to the location where the LVOTD measurement was taken, to obtain the LVOT time-velocity integral. Continuous wave Doppler signals were obtained in the same view. To calculate the stroke volume (SV), LVOT area (calculated from LVOTD) was multiplied by the LVOT velocity–time integral. The resulting SV was then normalized for body surface area (BSA) to obtain the indexed stroke volume (SVI). The aortic valve area (AVA) was calculated using the continuity equation and subsequently indexed to the BSA resulting in the indexed AVA (iAVA) [15]. MGs were derived by following the continuous-wave Doppler jet across the valve in various windows using the modified Bernoulli equation [16]. The Simpson’s biplane method was used to calculate the ejection fraction by estimating the end-diastolic volume (EDV) and the end-systolic volume (ESV) [3].

### Invasive gradient measurements

MG measurements were conducted immediately pre- and post- TAVI. Left ventricular pressure was recorded using a 6-French pigtail catheter positioned in the middle of the left ventricle (LV). Similarly, aortic pressure was measured in the ascending aorta using a separate pigtail catheter [6] The mean pressure gradient was calculated by integrating the differences in blood pressure between the ventricle and the aorta during systole [3].

### Study endpoints

The primary clinical endpoint was the discordance between invasive and non-invasive mean MGs before and after TAVI. The secondary clinical endpoint was defined as moderate or severe structural valve deterioration (SVD) at 1 month, and at 1 and 5 years. Moderate patient prosthesis mismatch (PPM) was defined as 0.65 cm^2^/m^2^ ≤ iAVA < 0.85 cm^2^/m^2^, while severe PPM was defined as iAVA ≤ 0.65 cm^2^/m^2^. Moderate SVD was defined as an increase in MGs by ≥ 10 mmHg from discharge MG as baseline, resulting in an MG ≥ 20 mmHg, or a decrease in AVA by ≥ 0.3 cm^2^ or ≥ 25%, moderate PPM, and moderate aortic regurgitation (AR; both paravalvular and transvalvular). Severe SVD was defined as an increase in transvalvular MG by ≥ 20 mmHg from discharge MG as baseline, resulting in an MG ≥ 30 mmHg with a concomitant decrease in EOA by ≥ 0.6 cm^2^ or ≥ 50%, PPM < 0.65 cm^2^/m^2^, and severe AR [9, 10, 17, 18]. Clinical endpoints were all-cause mortality and MACE defined as cardiac death, disabling stroke, and /or major vascular complications at 5 years [17]. Subgroup analyses were performed for the following groups: valve size < 25 mm or > 25 mm, balloon- versus self-expandable, and valve-in-valve procedures versus native annulus.

### Statistical analysis

The categorical variables were expressed as frequencies and percentages, while continuous variables were displayed as median values and interquartile range. The comparison of continuous variables was conducted using the Kruskal–Wallis Rank Sum Test. For categorical variables, Fisher’s exact test and Pearson’s Chi-squared test were employed. The discrepancies between pre- as well as post-interventional invasive and non-invasive MGs were investigated using linear regression and Pearson correlations. An analysis of variance (ANOVA) was performed to test for systematic differences in measurement agreement across invasive and non-invasive MGs for device-type and sinus-of-valsalva-diameter (SOVd). For SOVd, MGs were stratified into tertiles based on the 33rd and 66th percentiles as cut-off points, as previously done by Tomii et al. [19]. Survival analysis was performed to evaluate death and MACE at 5 years for patients with MG above and below 20 mmHg at discharge and those with no PPM versus patients with moderate and high PPM using the Kaplan–Meier procedure, with differences in survival tested using the Log-Rank (Mantel–Cox) test. Relative hazards for death and MACE at 5-year follow-up were evaluated based on invasive and non-invasive post-TAVI mean gradients. Both unadjusted and adjusted analyses were performed, with adjustments made for LVEF, age, device-type, device size and eGFR. A Cox proportional hazards model was used to estimate hazard ratios for both death and MACE. A *p*-value of < 0.05 was considered statistically significant. Data analysis was carried out using R Studio (Version 2023.06.0 + 421 for macOS, Posit Software, Boston, Massachusetts, USA; https://posit.co/), running R (version 4.2.3, released on March 15, 2023, R-Foundation, Vienna, Austria; http://www.r-project.org).

## Results

### Patient cohort and baseline characteristics

From September 2011 to February 2023, a total of 1480 consecutive patients with AS underwent TAVI and were prospectively enrolled in the registry. For this analysis, 1353 (91%) patients were available. In the overall cohort, 642 (50%) patients were female and mean age was 83 [IQR: 79, 86] years. Among all patients, 765 (59%) had dyslipidaemia, 368 (28%) diabetes, 1056 (81%) hypertension, 128 (10%) chronic obstructive pulmonary disease, 154 (12%) cerebrovascular disease, 736 (57%) coronary artery disease and 227 (18%) prior myocardial infarction.

### Baseline characteristics stratified by post-TAVI mean gradient

Post-TAVI non-invasive MG was < 10 mmHg in 767 (57%) patients, between 10 and 20 mmHg in 537 (40%) and > 20 mmHg in 49 (4%) patients. The patients with lower MGs post-TAVI were significantly older (83.4 [79.4, 86.8] years, vs 81.6 [78, 85.4] years, vs 82.7 [76.4, 85.8] years, *p* < 0.001), had a lower BMI (25.9 [23.1, 29] kg/m^2^, vs 27.0 [24.2, 30.5] kg/m^2^, vs 26.7 [24.3, 29.4] kg/m^2^, *p* < 0.001), lower eGFR (49 [36, 63] ml/min, vs 55 [40, 72] ml/min, vs 56 [45, 68] ml/min, *p* < 0.001) and higher STS Calculated risk of mortality assessment (3.4 [2.3, 5.6], vs 3.2 [2.1, 5.1], vs 3.0 [2.0, 5.1], *p* = 0.049). Patients with lower MGs had a significantly higher LVEF at baseline (55 [45, 61] % vs 59 [50, 63] %, vs 60 [50, 65]%, *p* = 0.005). There were no differences in the incidence of preconditions and ECG-characteristics between groups (Table 1).

**Table 1:** Patient baseline characteristics stratified by non-invasive mean gradients at discharge

Variable	*N*	Overall, N = 1353^*1*^	< 10 mmHg, N = 767^*1*^	10–20 mmHg, N = 537^*1*^	> 20 mmHg, N = 49^*1*^	*p* value^*2*^
*Basic characteristics*						
Age	1296	82.7 (78.9, 86.1)	83.4 (79.4, 86.8)	81.6 (78.0, 85.4)	82.7 (76.4, 85.8)	< 0.001
Sex	1296					0.7
Male		654 (50%)	377 (52%)	253 (49%)	24 (49%)	
Female		642 (50%)	354 (48%)	263 (51%)	25 (51%)	
BMI	1294	26.3 (23.5, 29.5)	25.9 (23.1, 29.0)	27.0 (24.2, 30.5)	26.7 (24.3, 29.4)	< 0.001
estimated GFR	1289	51 (38, 66)	49 (36, 63)	55 (40, 72)	56 (45, 68)	< 0.001
NYHA Classification	1260					
I		131 (10%)	64 (9.0%)	63 (13%)	4 (8.5%)	
II		478 (38%)	264 (37%)	195 (39%)	19 (40%)	
III		558 (44%)	326 (46%)	210 (42%)	22 (47%)	
IV		93 (7.4%)	58 (8.1%)	33 (6.6%)	2 (4.3%)	
STS adult Cardiac Surgery Risk	1296	3.3 (2.2, 5.4)	3.4 (2.3, 5.6)	3.2 (2.1, 5.1)	3.0 (2.0, 5.1)	0.049
Left ventricular ejection fraction	1199	57 (45, 63)	55 (45, 61)	59 (50, 63)	60 (50, 65)	0.005
*Preconditions*						
Dyslipidaemia	1296	765 (59%)	444 (61%)	296 (57%)	25 (51%)	0.2
Diabetes	1296	368 (28%)	211 (29%)	144 (28%)	13 (27%)	0.9
Hypertension	1296	1056 (81%)	600 (82%)	422 (82%)	34 (69%)	0.084
Chronic obstructive pulmonary disease	1296	128 (9.9%)	78 (11%)	44 (8.5%)	6 (12%)	0.3
Cerebrovascular Disease	1296	154 (12%)	96 (13%)	54 (10%)	4 (8.2%)	0.3
Coronary artery disease	1296	736 (57%)	431 (59%)	275 (53%)	30 (61%)	0.11
Myocardial infarction	1296	227 (18%)	139 (19%)	81 (16%)	7 (14%)	0.3
*ECG characteristics*						
Rhythm	1144					
Sinus		787 (69%)	405 (64%)	346 (75%)	36 (78%)	
Atrial fibrillation		270 (24%)	180 (28%)	81 (18%)	9 (20%)	
Paced rhythm		79 (6.9%)	45 (7.1%)	33 (7.1%)	1 (2.2%)	
Other		8 (0.7%)	6 (0.9%)	2 (0.4%)	0 (0%)	
Any AV-block	1047	201 (19%)	107 (18%)	84 (20%)	10 (24%)	0.6
Right or left bundle branch block	1101					> 0.9
LBBB		137 (12%)	76 (12%)	56 (13%)	5 (11%)	
RBBB		135 (12%)	77 (13%)	53 (12%)	5 (11%)	
No		829 (75%)	460 (75%)	334 (75%)	35 (78%)	
Percutaneous coronary intervention	1296	459 (35%)	277 (38%)	165 (32%)	17 (35%)	0.1

^1^Median (IQR); *n* (%)

^2^Kruskal–Wallis rank sum test; Pearson’s Chi-squared test; Fisher’s exact test

### Procedural characteristics

In the overall cohort, 1160 patients (90%) had an extrathoracic—(transfemoral) and 133 patients (10%) an intrathoracic (transapical or transaortic) access site. Self-expandable valves were used in 838 patients (65%), balloon expandable valves in 373 patients (29%), and mechanical expandable valves in 82 patients (6.3%). Balloon pre-dilation was performed in 1048 patients (81%) (Table 2).

**Table 2:** Patient procedural characteristics stratified by post-interventional mean gradient

Variable		*N*		Overall, *N* = 1353^1^		< 10, *N* = 767^1^		10–20, *N* = 537^1^		> 20, *N* = 49^1^		*p* value^2^
Access site		1296										< 0.001
Extrathoracic				1163 (90%)		680 (93%)		441 (85%)		42 (86%)		
Intrathoracic				133 (10%)		51 (7.0%)		75 (15%)		7 (14%)		
Implanted device		1353										< 0.001
Boston Scientific Family				280 (21%)		173 (23%)		101 (19%)		6 (12%)		
Edwards Sapien Family				373 (28%)		164 (21%)		196 (36%)		13 (27%)		
Medtronic Family				239 (18%)		174 (23%)		60 (11%)		5 (10%)		
Other				269 (20%)		131 (17%)		120 (22%)		18 (37%)		
SJM-portico				192 (14%)		125 (16%)		60 (11%)		7 (14%)		
Device size [mm]		810										< 0.001
≤ 25 mm				174 (21%)		46 (9.6%)		111 (37%)		17 (53%)		
> 25 mm				636 (79%)		433 (90%)		188 (63%)		15 (47%)		
Device type		1293										
Balloon-expandable				373 (29%)		164 (22%)		196 (38%)		13 (27%)		
Mechanical-expandable				82 (6.3%)		27 (3.7%)		44 (8.5%)		11 (22%)		
Self-expandable			838 (65%)		538 (74%)		275 (53%)		25 (51%)			
Balloon-valvuloplasty	1293		1049 (81%)		607 (83%)		410 (80%)		32 (65%)		0.005	

^1^*n* (%)

### Invasive versus non-invasive mean gradients

**Fig. 1:**
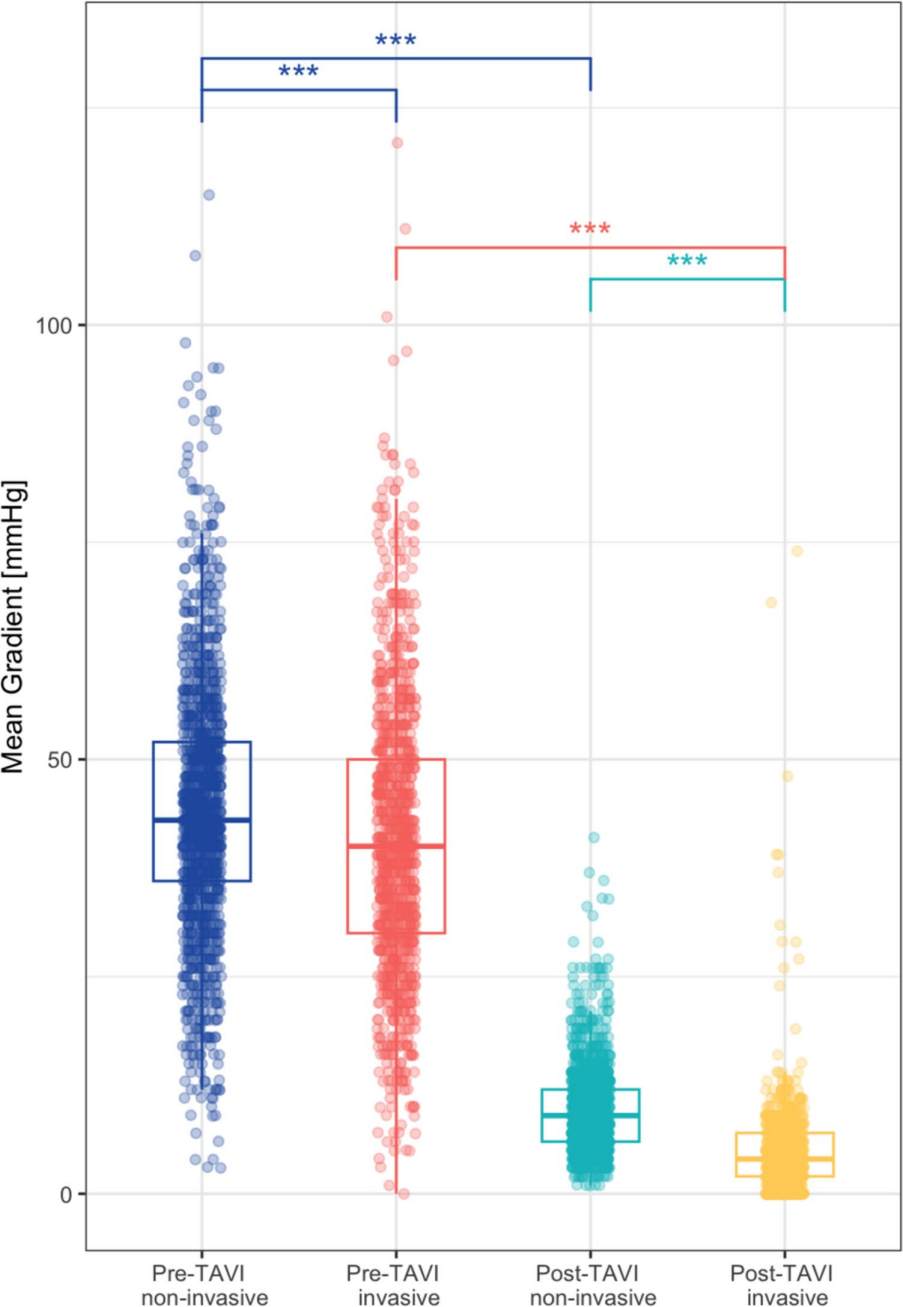
Discordance between non-invasive and invasive MGs pre- and post-TAVI (*p* < 0.001) as well as from pre to post-TAVI (*p* < 0.001). Data are presented as median and interquartile range. *MG* mean gradient, *TAVI* transcatheter aortic valve implantation; ***p < 0.001

**Fig. 2:**
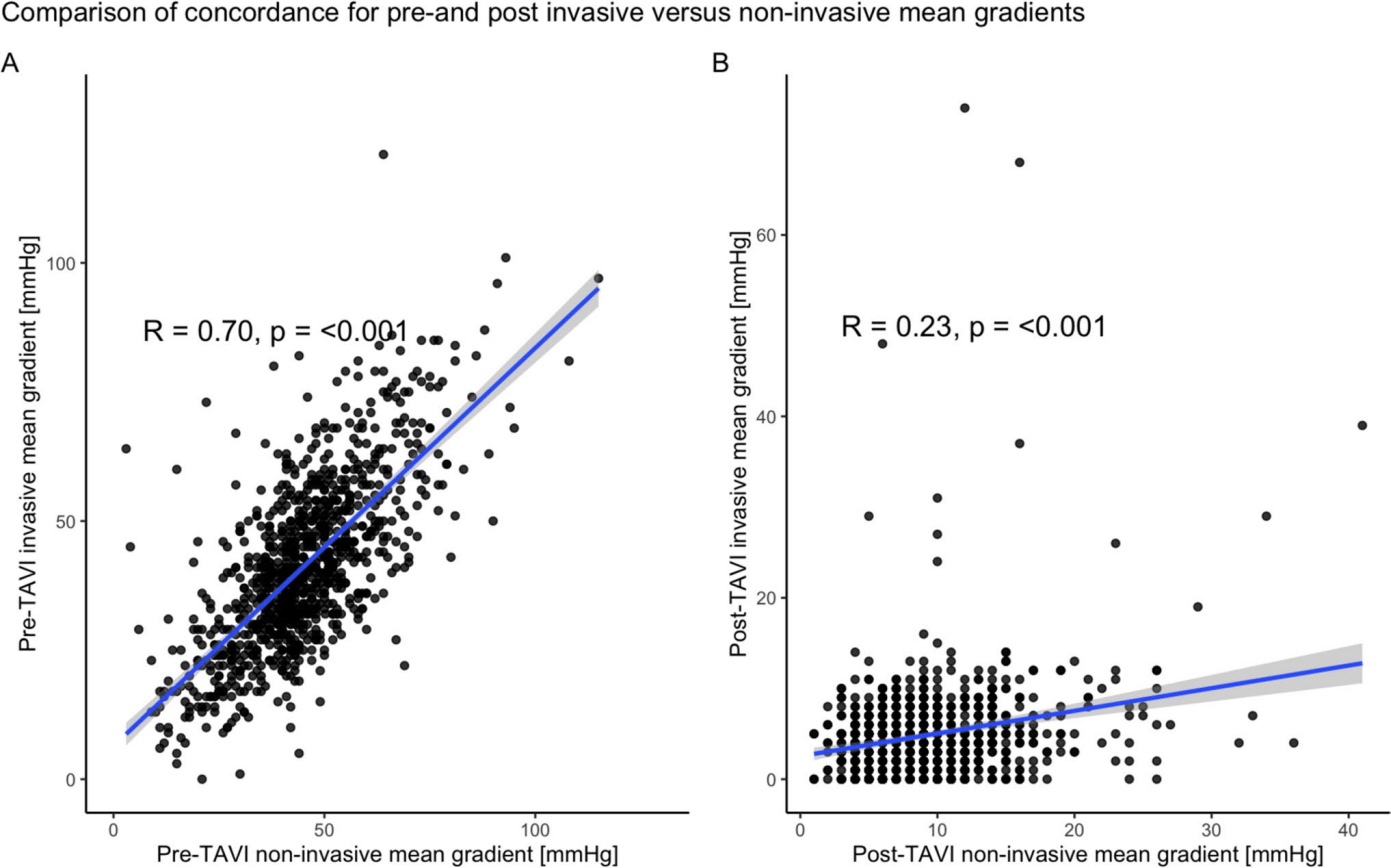

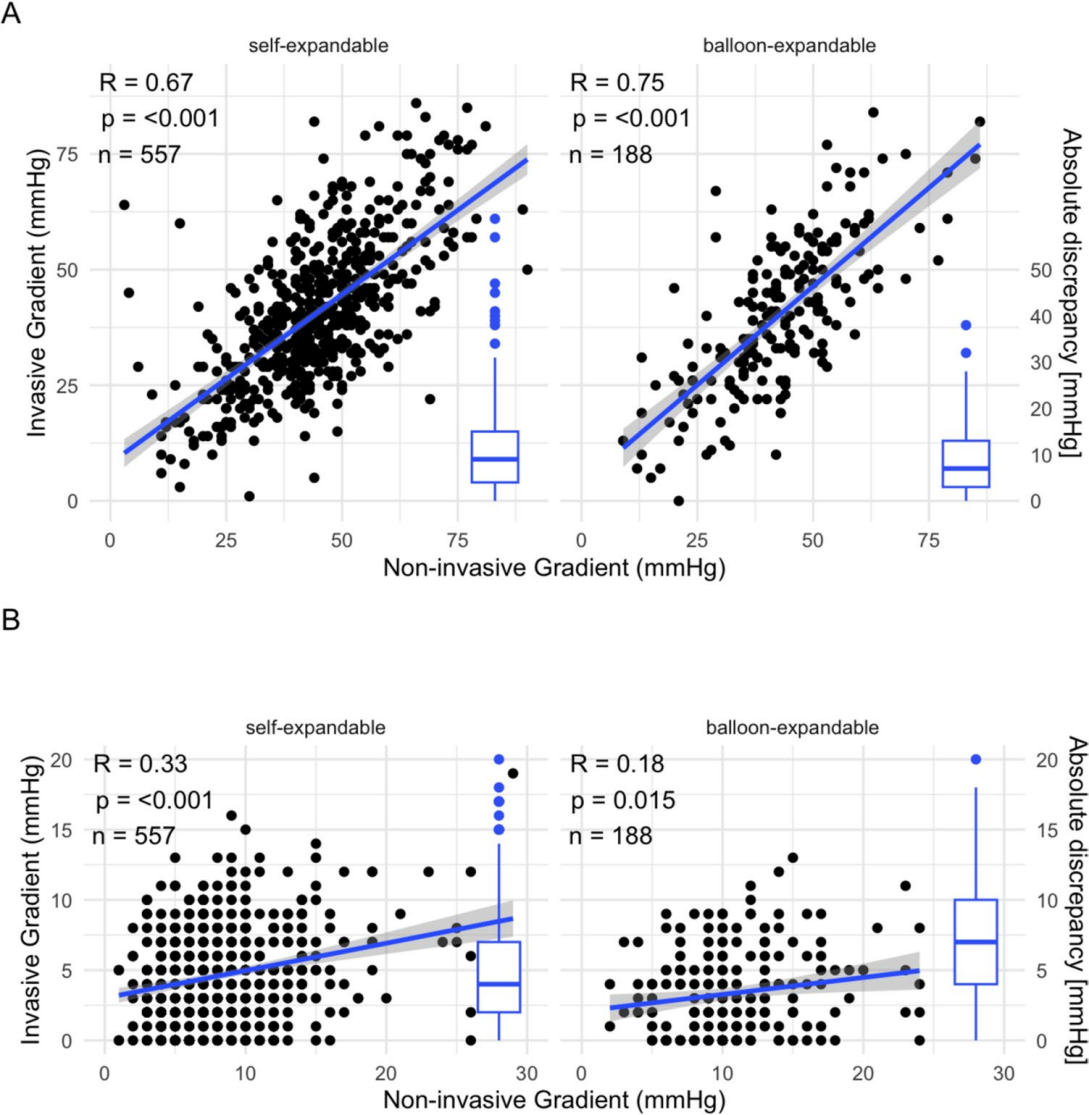

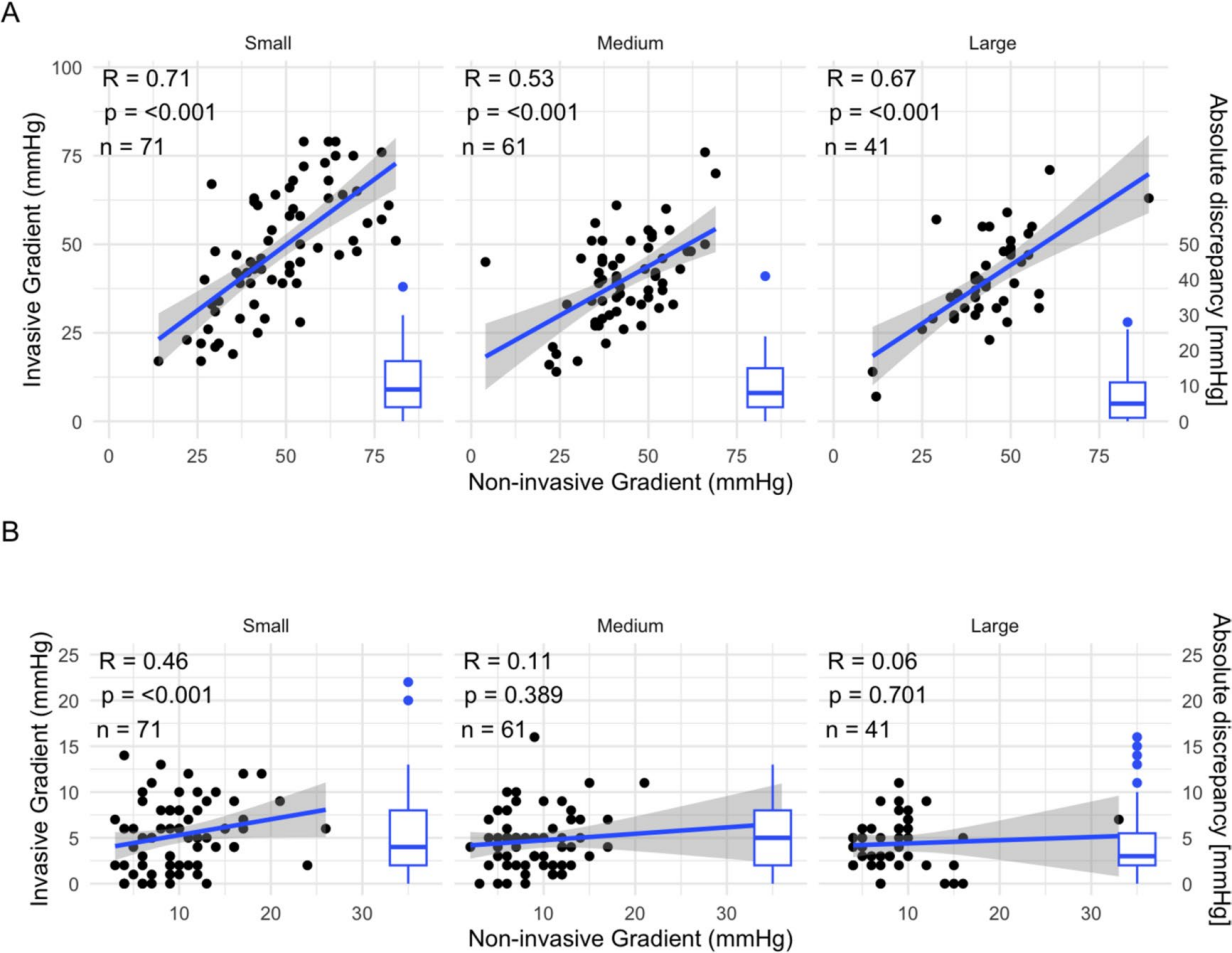
**a** Pearson correlation of concomitant invasive versus non-invasive MGs pre-TAVI (A) and post-TAVI (B). *MG* mean gradient, *TAVI* transcatheter aortic valve implantation. **b** Pearson correlation of concomitant invasive versus non-invasive MGs self- and balloon expandable valves, as well as corresponding distribution of absolute discrepancy of MGs pre-TAVI (A) and post-TAVI (B). *MG* mean gradient, *TAVI* transcatheter aortic valve implantation. **c** Pearson correlation of concomitant invasive versus non-invasive MGs for tertiles with small, medium and large SOVd, as well as corresponding distribution of absolute discrepancy of MGs pre-TAVI (A) and post-TAVI (B). *MG* mean gradient, *SOVd* sinus of valvalva diameter, *TAVI* transcatheter aortic valve implantation

At baseline, non-invasive MGs were significantly higher compared to invasive MGs (43 [36, 52] mmHg versus 40 [30, 50] mmHg, *p* < 0.001), with a mean difference of 5 [− 3, 11] mmHg. After TAVI, non-invasive MGs remained higher compared to invasive MGs (9 [6, 12] mmHg versus 4 [2, 7] mmHg, *p* < 0.001) with a mean difference of 5 mmHg [1, 8]. MGs decreased significantly both non-invasively (from 43 [36, 52] mmHg to 9 [6, 12] mmHg, *p* < 0.001), as well as invasively (from 40 [30, 50] mmHg to 4 [2, 7] mmHg, *p* < 0.001), respectively (Fig. 1). The non-invasive and invasive MGs showed a strong correlation pre-TAVI (*r* = 0.70, *p* < 0.001) but a weak correlation post-TAVI (*r* = 0.23, *p* < 0.001) (Fig. 2a). There was no difference in the concordance pre-interventionally between patients receiving self- or balloon expandable valves. Post-interventionally, however, patients receiving a self-expandable valve showed a significantly lower discrepancy and a much higher Pearson correlation between non- and invasive MGs (invasive–non-invasive post-interventional − 3.69 ± 5.1 vs − 7.47 ± 5.0, *p* < 0.001 and Pearson correlation *R* = 0.33, *p* < 0.001 vs *R* = 0.75, *p* = 0.015) (Fig. 2b).

Discrepancies between non-invasive and invasive MG did not differ between levels of SOVd. (pre-interventional *p* = 0.166, post-interventional *p* = 0.998) However, lower values of SOVd showed better Pearson correlation between invasive and non-invasive measurements of MG (small: *R* = 0.46, *p* < 0.001; medium: *R* = 0.11, *p* = 0.389; large: *R* = 0.06, *p* = 0.701) (Fig. 2c).

### Non-invasive mean gradients over time

**Fig. 3:**
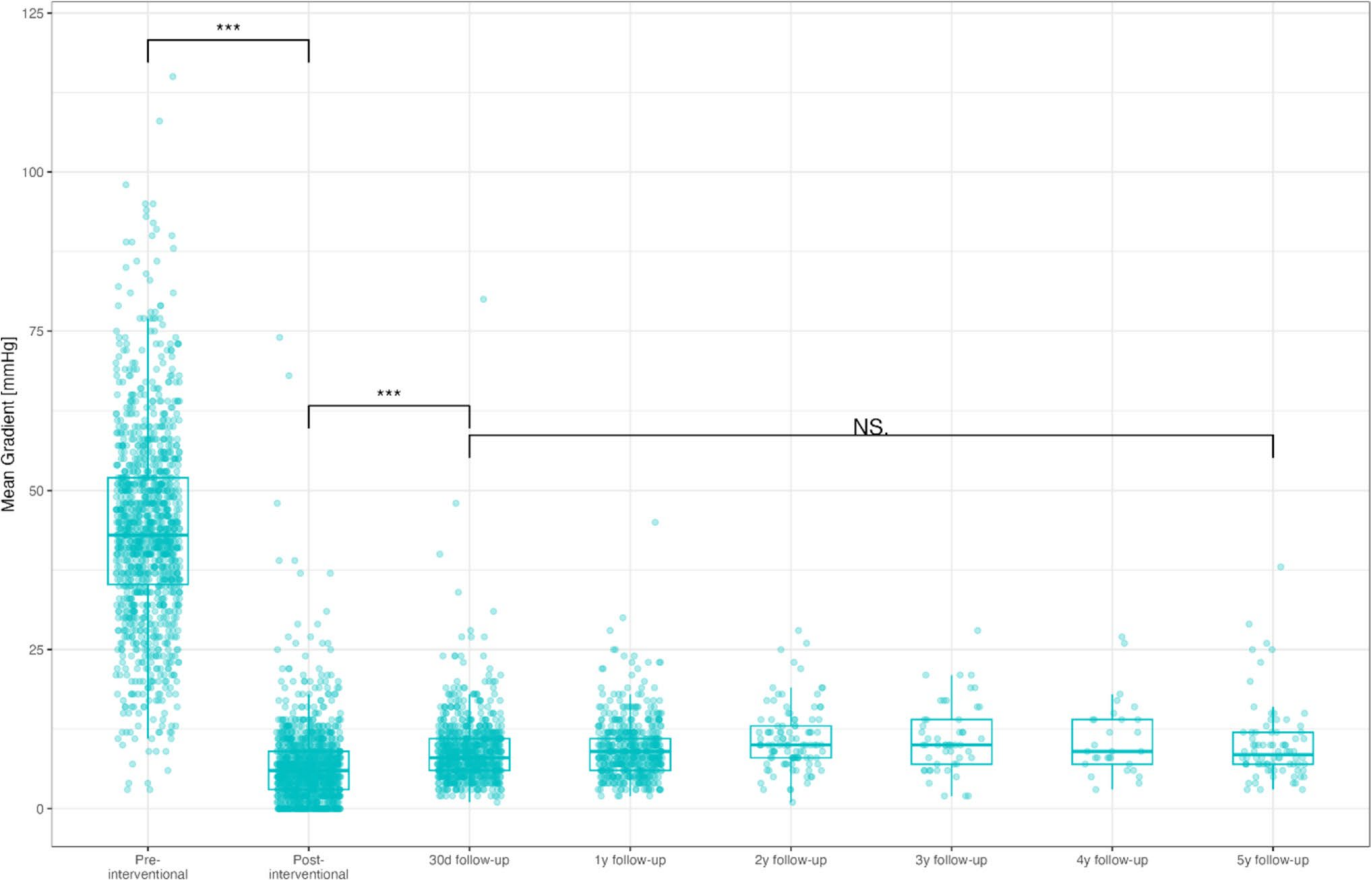
The progression of non-invasive MGs through 5 years. Data are presented as median and interquartile range. *MG* mean gradient, *NS*. not significant; ***p < 0.001

After one month, MGs remained constant with a mean of 8 [6, 11] mmHg and remained stable through 5 years. (Fig. 3) There was no difference in MG between self- or balloon-expandable valves over 5 years (Supplemental Fig. 1A). However, MGs at 1 month were lower in patients who received extrathoracic versus intrathoracic access (8 [6, 11] mmHg versus 10 [8, 13] mmHg, *p* < 0.001) and remained significantly lower during subsequent follow-ups (Supplemental Fig. 1B) In addition, smaller valves (≤ 25 mm) showed higher MGs during follow-up at one month compared to larger valves (> 25 mm) (11 [8, 14] mmHg versus 7 [6, 10] mmHg, *p* < 0.001), which remained significantly elevated throughout the observed follow-up period.

### Structural valve deterioration and patient prosthesis mismatch

**Fig. 4:**
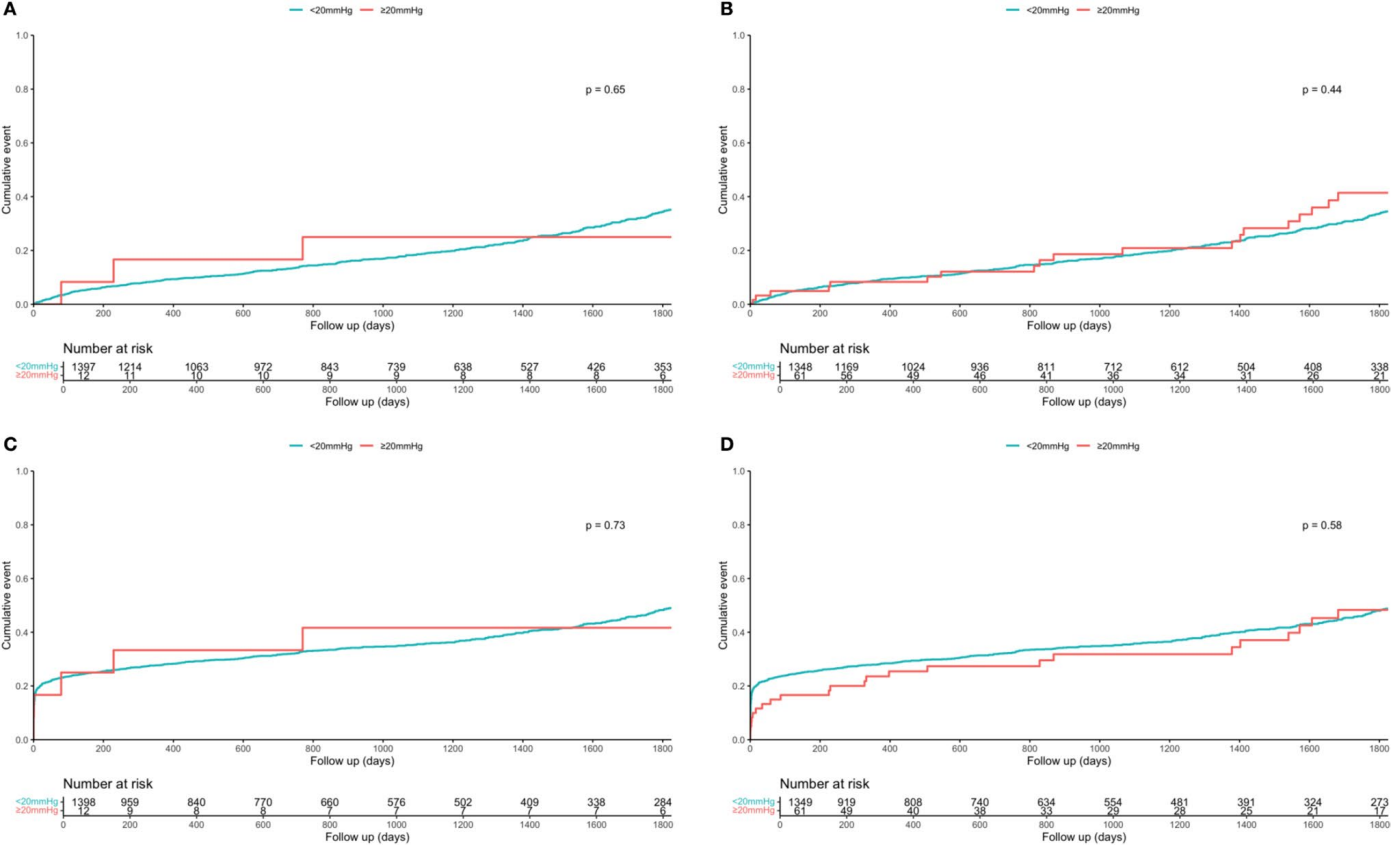
Kaplan–Meier curves for 5-year follow-up regarding Mortality for invasive (**A**) and non-invasive (**B**) post-interventional gradients above and below 20 mmHg, as well as regarding major adverse cardiac events (MACE) for invasive (**C**) and non-invasive (**D**) post-interventional gradients above and below 20 mmHg. *MG* mean gradient

Moderate valve deterioration was present in 28 patients (3.2%) at 1 month, in 23 patients (3.9%) at 1 year, and in 12 patients (11%) at 5 years, respectively. Severe valve deterioration was documented in 6 patients (0.7%) at 1 month, in 3 patients (0.5%) at 1 year, and 1 patient (0.9%) at 5 years, respectively. Neither invasive nor non-invasive gradient > 20 mmHg post-intervention were associated with higher mortality compared to below 20 mmHg (25% versus 23%, *p* = 0.65 and 31% versus 23%, *p* = 0.44) or MACE at 5 years (41% versus 39%, *p* = 0.73 and 39% versus 39%, *p* = 0.58). (Fig. 4).

**Fig. 5:**
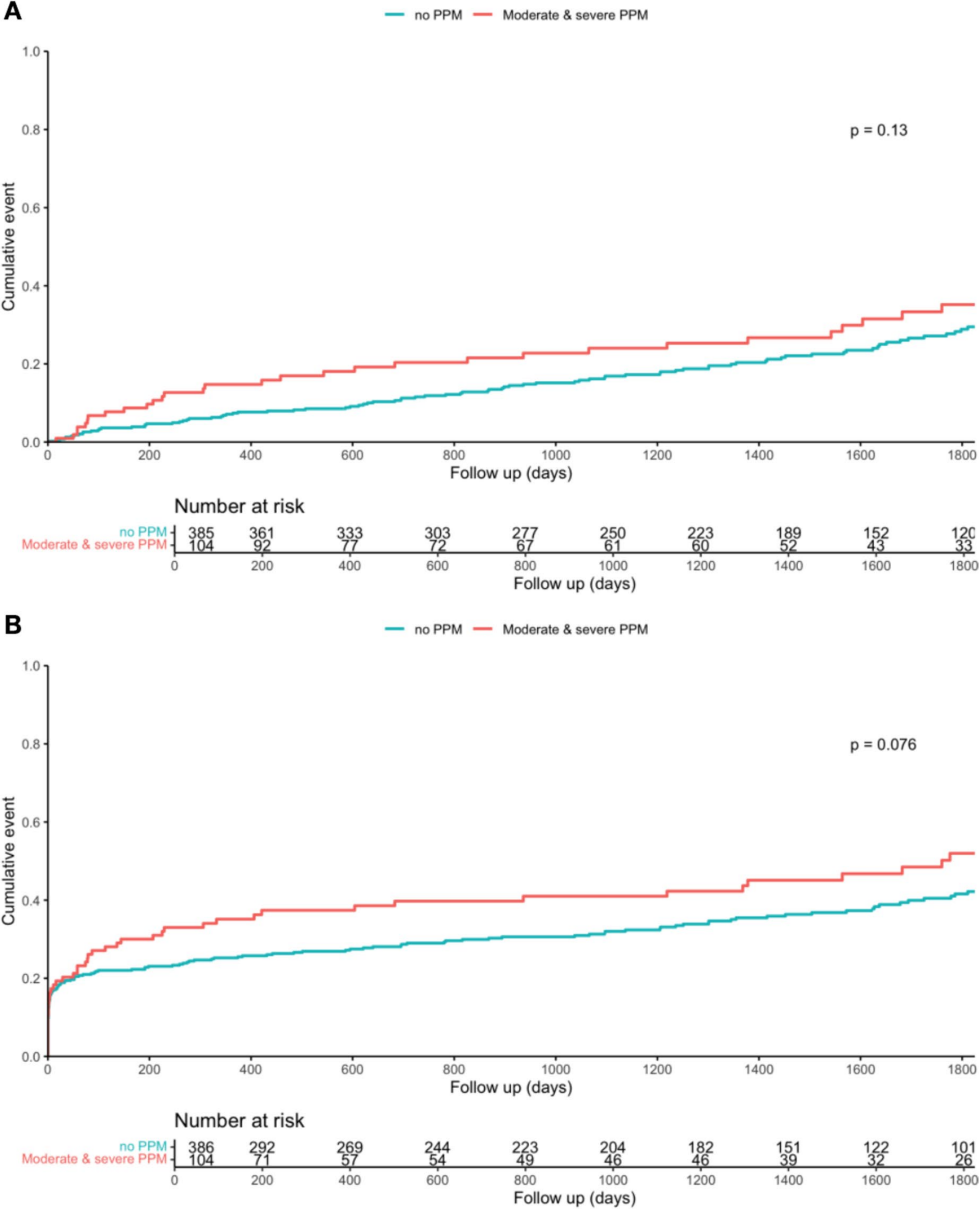
Kaplan–Meier curves for 5-year follow-up between patients with severe and moderate post-TAVI patient-prosthesis mismatch (PPM) (indexed aortic valve area < 0.85 cm^2^/m^2^) and those without regarding mortality (**A**) and major adverse cardiac events (MACE) (**B**)

Moderate PPM was present in 78 patients (7.8%) after TAVI, in 63 patients (5.7%) at 1 month, in 29 patients (2.8%) at 1 year, and in 5 patients (1.2%) at 5 years. Severe PPM was present in 26 patients (2.7%) after TAVI, in 37 patients (3.4%) at 1 month, in 20 patients (1.9%) at 1 year and in 8 patients (1.9%) at 5 years, respectively. Moderate and severe as compared with lower levels of PPM following TAVI had numerically higher rates of mortality and MACE at 5 years. However, these differences were not statistically significant (29% vs. 22%, *p* = 0.13 for mortality; 46% vs. 37%, *p* = 0.08 for MACE). (Fig. 5A, B).

### Outcomes

**Fig. 6:**
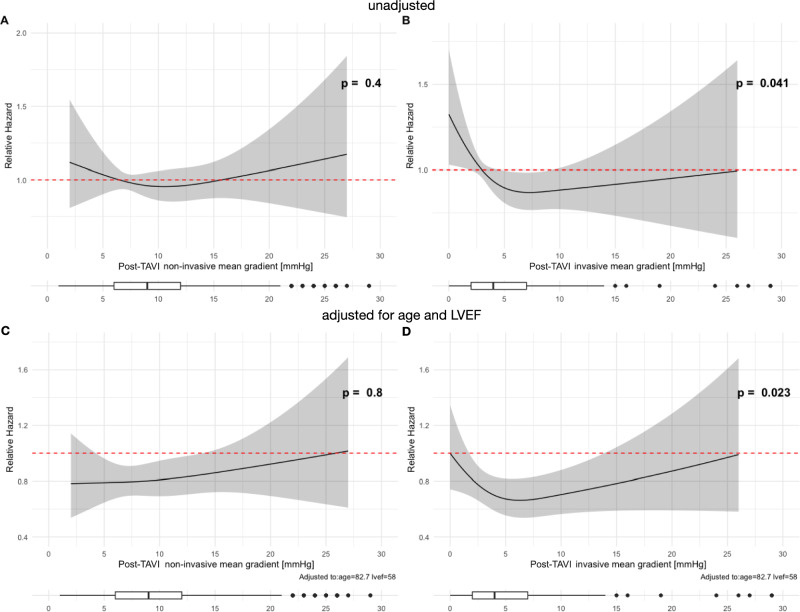
Relative hazard for mortality at 5-year follow-up for post-TAVI non-invasive mean gradients (left: **A, C**) and invasive mean gradients (right: **B, D**) unadjusted, and adjusted for left ventricular ejection fraction and age. *LVEF* left ventricular ejection fraction, *MG* mean gradient, *TAVI* transcatheter aortic valve implantation

At the 5-year follow-up, the relative hazard for mortality was evaluated based on invasive and non-invasive post-TAVI MGs, both with and without adjustments for age, LVEF, device type,—size and renal function. For non-invasive MGs, neither the unadjusted nor the adjusted analysis revealed significant associations with the relative hazard for mortality (*p* = 0.4 and *p* = 0.8) (Fig. 6A, C). In contrast, for invasive MGs, both analyses revealed a significant association (*p* = 0.041 and *p* = 0.038) (Fig. 6B, D).

**Fig. 7:**
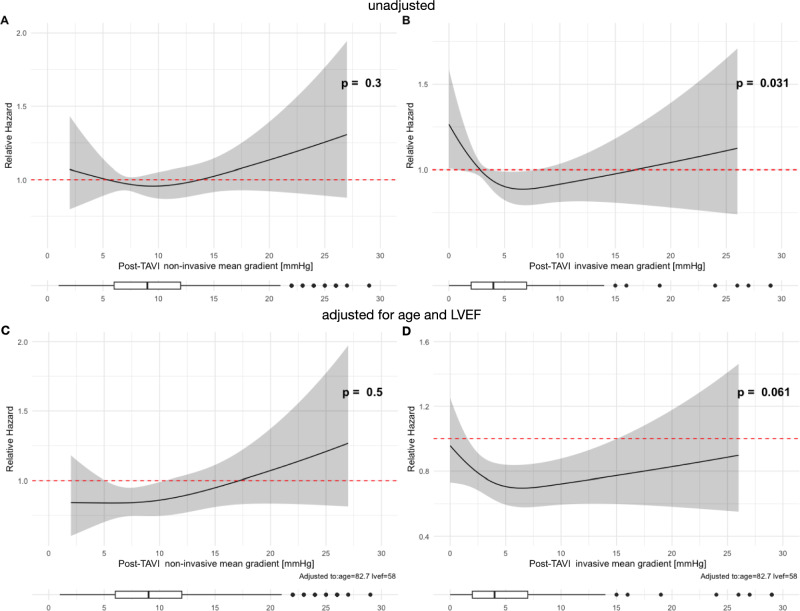
Relative hazard for major adverse cardiac events (MACE) at 5-year follow-up for post-TAVI non-invasive mean gradients (left: **A, C**) and invasive mean gradients (right: **B, D**) unadjusted, and adjusted for left ventricular ejection fraction and age. *LVEF* left ventricular ejection fraction, *MG* mean gradient, *TAVI* transcatheter aortic valve implantation

Similarly, the relative hazard for MACE at the 5-year follow-up was assessed based on invasive and non-invasive post-TAVI MGs with and without adjustments for age, LVEF, device type,—size and renal function. For non-invasive MGs, neither the unadjusted nor the adjusted analysis showed significant association with the relative hazard for MACE (*p* = 0.3 and *p* = 0.5) (Fig. 7A, C). In contrast, for invasive MGs, both the unadjusted and adjusted analysis revealed a significant association (*p* = 0.031, *p* = 0.033). (Fig. 7B, D). Very low post-TAVI invasive MGs were associated with excess mortality and MACE. MGs around 5–10 mmHg led to a lower relative hazard, which increased with higher MGs. This increase in hazard was particularly notable for mortality, even after adjusting for age and LVEF.

## Discussion

In this large study, we report five major findings. First, both pre- and post-procedural invasive MGs were lower than non-invasive MGs, with pre-procedural MGs showing a stronger correlation than post-procedural MGs, with significant differences between self- and balloon-expanding valves but not sinus of valsalva diameter. Second, non-invasive MGs at discharge were lowest and increased until 30-day follow-up, but remained constant for the rest of the follow-up period through 5 years. Third, post-procedural invasive and non-invasive MGs > 20 mmHg were not predictive of either mortality or MACE during follow-up. Fourth, VARC-3 defined moderate and severe SVD were documented at 30-day follow-up in 28 (3.2%) and 6 patients (0.7%), respectively. Moderate and severe PPM were present in 63 (2.8%) and 37 (3.1%) patients at 30-day follow-up. Neither SVD (defined as MG at discharge ≥ 20 mmHg) nor PPM were associated with adverse outcomes. Fifth, very low and higher invasive, but not non-invasive MGs were related to adverse outcomes.

This study corroborates and extends prior findings in assessing aortic valve stenosis and prosthesis valves both invasively and non-invasively. The overestimation of non-invasive MGs compared to invasive MGs is a well-described phenomenon [6, 20] This discrepancy can be attributed to an inherent limitation of the Bernoulli equation, which is used for normally functioning prosthesis. The Bernoulli equation reflects aortic velocity, which is influenced by various factors and does not necessarily represent the true valve gradient [7, 21]. This phenomenon, called pressure recovery, reflects the fact that some of the velocity generated in flow across the valve is reconverted back to pressure energy in the proximal aorta without a true loss in energy from the stenosis. The high velocity across the valve does not completely reflect energy lost during systole and this portion not lost is then recovered as pressure distally in the aorta. The pressure difference between the left ventricular pressure and that “recovered” aortic pressure best reflects true energy loss during ejection and valvular stenosis burden. In contrast, echo‐Doppler picks up the highest gradient before this pressure recovery and overestimates the true degree of stenosis/energy loss. Such pressure recovery is exacerbated in the setting of high flow states, small noncompliant aortas, and geometric alterations where there is not the normal outward turbulent expansion of blood past the valve in the sinuses [22]. The factors that have previously been shown to influence pressure recovery and thereby differences in MG are valve-type, due to the effect self- and balloon-expandable valves have on flow-shape, and SOVd, with smaller SOVd resulting in more laminar flows and better Doppler alignment [23, 24]. Previous studies have shown self-expandable valves to offer a superior hemodynamic profile, possibly due to positional differences [25, 26]. Our results align with and expand on these findings in revealing less discrepancy between invasive and non-invasive post-interventional MGs (− 3.69 ± 5.1 vs − 7.47 ± 5.0, *p* < 0.001) and higher correlation between the MGs (*R* = 0.33, *p* < 0.001 vs *R* = 0.75, *p* = 0.015), suggesting better pressure recovery in self-expandable valves. Furthermore, smaller SOVd showed better correlation of MGs, even though there was no difference in absolute discrepancy between the tertiles.

The stronger correlation for MGs pre- to post-procedurally might in part be related to the relatively higher values pre-procedurally and the broader range of values in for both invasive and non-invasive values.

The lowest values of non-invasively derived MGs were observed at discharge and tended to increase until one-month post-procedure. This finding is consistent with current literature [7, 27, 28]. No significant trend was observed over the following 5 years, which is in line with other studies [29]. Nonetheless, other studies have found an incremental increase in gradient over one to four years, although their number of patients at later follow-ups was limited [30].

Furthermore, patients discharged with a non-invasive MG > 20 mmHg did not exhibit higher mortality compared to patients with MGs < 20 mmHg. However, the significance of this finding might be limited due to the small number of patients exhibiting high MGs. There was no significant difference in survival or the incidence of MACE across different invasively derived values. In our study, neither variable was adequate for predicting outcomes. The relationship between post-TAVI MGs and clinical outcomes remains contentious. Several studies indicate improved survival with lower MGs compared to intermediate groups [31]. However, the consensus in the literature suggests that a high MG is not a predictor of worse outcomes [31–33]. Various explanations have been proposed for the association between lower MGs and reduced mortality rates. For instance, untreated AS may lead to a progressive decline in myocardial contractility, subsequently lowering MG [34]. Further research indicates that MG is a limited measure of AS severity, as it depends on accurate velocity data, which is flow-dependent and requires parallel alignment of the ultrasound beam [3].

There was no significant correlation between the severity of structural valve deterioration (SVD), as measured both invasively and non-invasively post-TAVI and at discharge, and the incidence of mortality or MACE. These findings align with previous studies [35, 36]. However, conflicting evidence exists. A recent study reported a significant increase in 5-year mortality and MACE among patients developing SVD [37]. Similarly, another indicated that SVD, which is common during the first 12 months after TAVI, is a predictor of higher mortality [38].

Moderate and high PPM were not associated with worse outcome. Some studies confirm that high PPM is not necessarily linked to higher mortality, particularly when high PPM is accompanied by normal left ventricular ejection fraction (LVEF) rather than low flow [32, 33]. The same study also suggests that the conflicting impact on mortality may be related to various underlying comorbidities that affect flow and LVEF. Therefore, it is questionable whether PPM alone is an adequate predictor of outcomes [39]. The studies that have shown an association between PPM and increased risk of death were often limited by their small sample sizes, which could explain these discrepant findings [28, 40, 41, 43]. Further studies with larger cohorts are warranted to investigate this further.

Our results suggest that invasive MGs may be a more reliable predictor of long-term outcomes post-TAVI compared to non-invasive measures. In recent years, the patients with low-flow, low-gradient (LFLG) AS have been extensively described and shown to have significantly worse outcomes and were associated with more pronounced cardiac remodelling, hemodynamic challenges and left ventricular dysfunction [26, 42]. Furthermore, consistent with our findings, previous studies have indicated that both very low and high post-interventional MGs are associated with an increased relative risk of mortality and MACE. For example, Khalili et al. emphasized the prognostic value of invasive hemodynamic measurements, even after adjustment for STS-Score, LVEF, and indexed stroke volume. Their study identified an optimal gradient range of 5–10 mmHg, which associates with lower relative hazards [31]. Our study corroborates this cut-off as predictive for mortality. Similarly, Didier et al. reported that patients with higher invasive MGs post-TAVI experienced significantly higher rates of adverse events and mortality, aligning with our observed gradient-mortality relationship [27].

We acknowledge the following limitations of our analysis: although the registry included a large number of patients, some subgroups, particularly those used for comparing post-interventional MGs on outcomes, were small, which may lead to non-generalizable assumptions. Additionally, the data are derived from a single-centre registry, possibly limiting generalizability. The echocardiographic data are user-dependent and can be over- or underestimated. However, our cohort provides a robust representation of real-world data and findings.

In conclusion, invasive as compared with non-invasive MGs correlated better before than after valve implantation, whereas invasive MGs were always lower than non-invasive MGs. Lower invasive MGs after TAVI appeared to be associated with favourable long-term outcomes.

## Conflict of interest

Thomas Nestelberger has received research support from the Swiss National Science Foundation (P400PM_191037/1), the Prof. Dr. Max Cloëtta Foundation, the Margarete und Walter Lichtenstein-Stiftung (3MS1038), and the University Hospital Basel as well as speaker/consulting honoraria or research support from Edwards Lifesciences, Pronova Medical, Meril, Boston Scientific, Medtronic, Abbott, Beckman Coulter, Bayer, Ortho Clinical Diagnostics and Orion Pharma, outside the submitted work. All other authors declare that they have no conflict of interest with this study. All authors critically reviewed the manuscript and approved the final version for submission. The manuscript and its contents have not been published previously and are not being considered for publications elsewhere in whole or in part in any language, including publicly accessible web sites or e-print servers.

## Supplementary Information

Below is the link to the electronic supplementary material.

Supplementary file1 (DOCX 235 KB)

## Data Availability

The data that support the findings of this study are available from the corresponding author upon reasonable request.

## Abbreviations

AVA: Aortic valve area
BSA: Body surface area
GFR: Glomerular filtration rate
iAVA: Indexed aortic valve area
LVEF: Left ventricular ejection fraction
LVOT: Left ventricular outflow tract
LVOTD: LVOT diameter
MACE: Major adverse cardiovascular events
MG: Mean pressure gradient
PG: Peak pressure gradient
PIMG: Post-TAVI invasive mean gradient
PPM: Patient prosthesis mismatch
SV: Stroke volume
SVI: Indexed stroke volume
TAVI: Transcatheter aortic valve implantation
TEE: Transoesophageal echocardiography
TTE: Transthoracic echocardiography
