# Supplementary material for: Comparison of invasive and non-invasive gradients before and after TAVI and their implications on clinical outcomes
Source: Cardiovasc Interv Ther. 2024 Dec 19;40(2):362–77. doi: 10.1007/s12928-024-01074-6 (PMC11910411; doi:10.1007/s12928-024-01074-6)
Supplement: Supplementary file 1 — Supplementary file1 (DOCX 235 KB) [file 12928_2024_1074_MOESM1_ESM.docx]

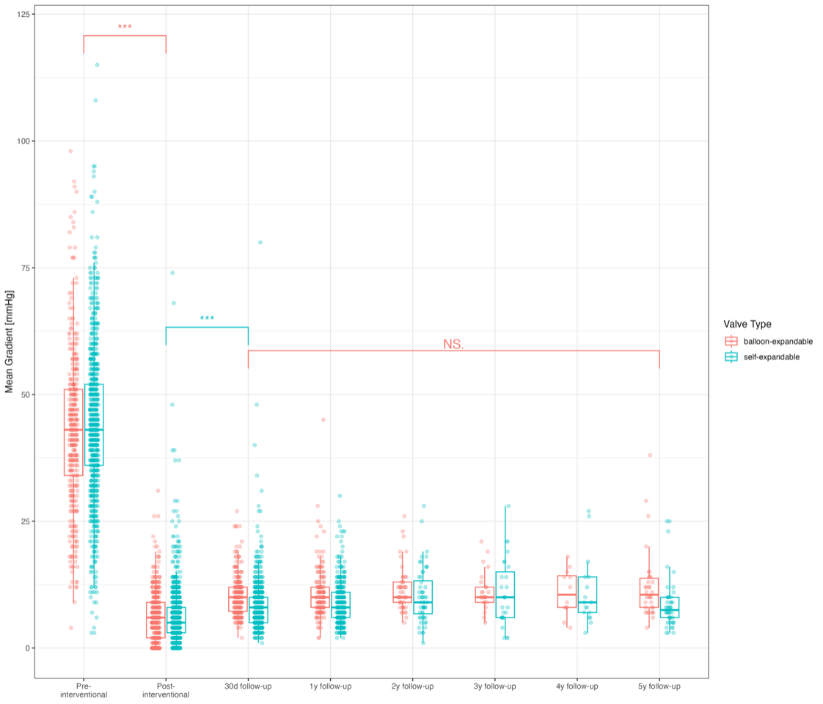


Supplemental Figure 1A. The progression of non-invasive MGs through 5 years between patients receiving balloon and self-expandable valves. Data are presented as median and interquartile range.
MG = mean gradient; NS.= not significant; ***: p < 0.001


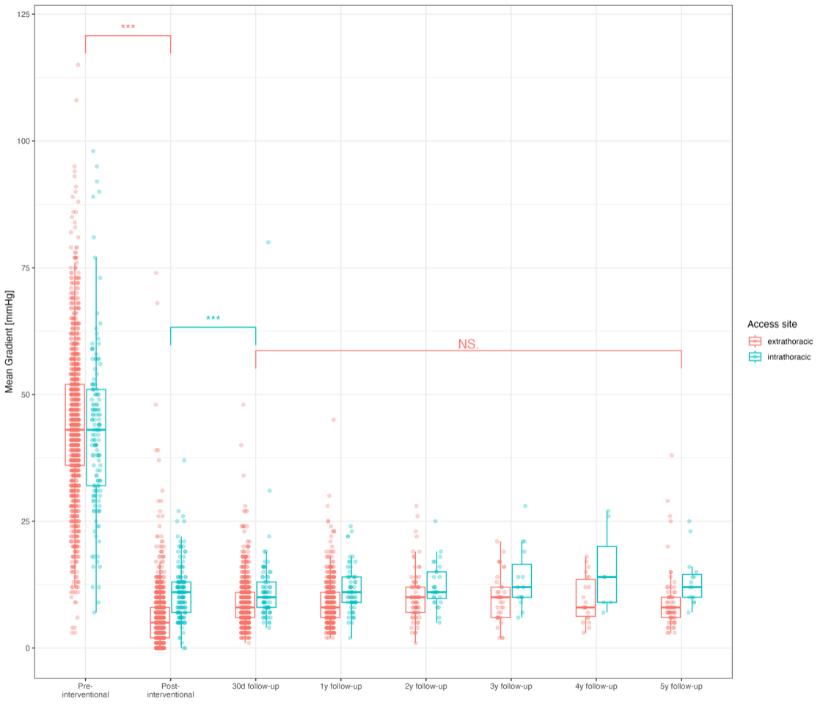


Supplemental Figure 1B. The progression of non-invasive MGs through 5 years between patients undergoing intra- versus extrathoracic access. Data are presented as median and interquartile range.
MG = mean gradient; NS.= not significant; ***: p < 0.001

Supplemental Table 1: Multivariable analysis with adjustment for post-interventional invasive mean gradient, age, left ventricular ejection fraction, device type, - size and renal function

| **Multivariable (adj. for post-interventional invasive mean gradient, age, LVEF, device type, device size, eGFR)** | | | | |
| --- | --- | --- | --- | --- |
|  | **HR (95% CI)** | **p** | **HR (95% CI)** | **p** |
|  | **Death, 5y^1^** | | **Major adverse events, 5y^1^** | |
| Post-TAVI invasive MG (0-4mmHg) | 0.91 (0.82 - 1.01) | 0.042 | 0.76 (0.61 – 0.94) | 0.013 |
| Post-TAVI invasive MG (>4mmHg) | 1.10 (1.01 – 1.20) | 0.038 | 3.08 (1.80-8.06) | 0.022 |
| Age | 1.00 (0.95 - 1.06) | 0.126 | 0.98 (0.94 - 1.03) | 0.054 |
| LVEF | 0.96 (0.94 - 0.98) | 0.002 | 0.96 (0.94 - 0.98) | 0.001 |
| Device Type | 0.61 (0.42 - 0.86) | 0.006 | 0.81 (0.58 - 1.11) | 0.191 |
| Device Size | 1.06 (1.00 - 1.12) | 0.072 | 1.03 (0.98 - 1.08) | 0.281 |
| eGFR | 0.99 (0.97 - 1.01) | 0.011 | 0.99 (0.98 - 1.01) | 0.039 |
| ^1^Cox-regression model with adjustment for post-interventional invasive mean gradient (MG), age, left ventricular ejection fraction, device type, - size and renal function | | | | |
